# Premarital sexual intercourse and associated factors among adolescent students in Debre-Markos town secondary and preparatory schools, north west Ethiopia, 2017

**DOI:** 10.1186/s13104-019-4132-4

**Published:** 2019-02-20

**Authors:** Geremew Kindie Behulu, Kiber Temesgen Anteneh, Getie Lake Aynalem

**Affiliations:** Department of Midwifery, College of Medicine and Health Science, University of Gondar, Gondar, Ethiopia

**Keywords:** Premarital sexual practice, School adolescents, Ethiopia

## Abstract

**Objective:**

To assess the magnitude and factors associated with premarital sexual intercourse among adolescent students of the secondary and preparatory school in Debre-Markos town, northwest Ethiopia, 2017.

**Results:**

Among secondary and preparatory school adolescent students, 31.3% reported pre-marital sexual intercourse. This shows that premarital sexual intercourse among secondary and preparatory school adolescents is high. Significantly associated factors were: being male (AOR = 1.9, 95% CI 1.21, 2.93), having pocket money (AOR = 3.1, 95% CI 2, 4.81), adolescents who did not discuss sexual issue with close friends (AOR = 8.6, 95% CI 5.27, 13.91) and peer pressure (AOR = 7.7, 95% CI 3.73, 15.69).

## Introduction

Premarital sex is penetrative vaginal intercourse performed between couples before formal marriage [1, 2]. World Health Organization (WHO) defines adolescent as persons between the age group of 10–19 years old [3]. Adolescence is the period of transmission from childhood to maturity and is characterized by spurt of physical, mental, emotional, social and psychosexual development [4].

Adolescents are a growing and larger segment of the population of developing countries and an estimated 1.2 billion young people in the world, 85% live in developing countries [5]. Nearly 85% of the world’s adolescent population live in developing countries and in some sub-Saharan countries, population below 15 years of age is five times greater than the population over 55 years of age [6].

The adolescent years are the time of rapid growth, exploration, and risk-taking. In many countries, an average of 29% of boys and 23% of girls are sexually active including premarital sex [7].

Many adolescents face pressures to use alcohol, cigarettes, or other drugs and to initiate sexual relationships at earlier ages, to put themselves at high risk for intentional and unintentional injuries and risky sexual behaviors [8]. Females, particularly adolescent girls may end up with unwanted pregnancies, abortions, teenage deliveries, and various complications of these including death. Moreover, the girls may drop out from school to look after their children, and in most cases, they become economically reliant on upon their parents [1].

Nearly 70% of premature deaths among adults can be linked to behaviors that were initiated during adolescence [9]. Unwanted pregnancy can be associated with higher likelihood of early motherhood, unsafe abortion, and other pregnancy-related complications [10].

Several studies in sub-Saharan Africa have also documented high and increasing premarital sexual activities among adolescents [8]. According to EDHS 2016, 13 percent of women age 15–19 in Ethiopia have begun childbearing [11]. In Amhara region, pre-marital sexual debut was reported as early as 12 to 13 years [10].

Studies have documented that early sexual initiators were more likely to report undesired consequences of sexual initiation such as teenage motherhood, not using a condom at first sex and sexually transmitted infections (STIs). Adolescents are also likely to have an intimate partner who is five or more years older and be involved in multiple sexual partnerships [12].

Even though some studies were conducted on this topic, most of them focused on only females [5, 10, 13], youth [14, 15] and university students [7, 8], there is a gap of including males, married females and adolescents. Therefore, this study tried to fill the above gaps.

## Main text

### Methods

#### Study design and setting

An institutional based cross-sectional study design was conducted among secondary and preparatory school adolescent students in Debre-Markos town from November 23–27, 2017. The town is located at about 295 km to the capital city of Ethiopia. Based on the 2007 national census conducted by the Central Statistical Agency of Ethiopia (CSA), this town has a total population of 62,497, of whom 29,921 were men. The majority Ethiopian Orthodox Christianity followers, with 97.03% reporting [16]. There are three secondary and two preparatory schools in the town.

#### Sample size and sampling procedure

A single population proportion formula was used to calculate the sample size of 624 by taking the following assumptions. From the previous study conducted in Jimma town on premarital sexual practice among school adolescents [17], 25.27%, 95% CI, 5% marginal error and n = ((Zα/2)^2^ p (1 − p))/W^2^. Adding a 15% non-response rate and design effect of 2, the total sample size required was 624.

Multi-stage stratified sampling technique used to select adolescent students. All regular adolescent students (secondary and preparatory) attending class at the time of the survey in Debre-Markos secondary and preparatory school divided into different strata. Grade considered as strata. The number of adolescent students from each grade level and sections according to their sex identified by using the name and sex list of each section.

#### Operational definitions

*Age at initial sexual contact* is age at first intercourse (vaginal–penile penetration).

*Early sexual initiation* was taken as an experience of first intercourse before 18 years of age.

*Sexually active* A student who had a penetrative sexual intercourse (vaginal) at least once prior to the study.

*Peer pressure* when the individual said yes/no to question saying “did your friend initiate you to do sex?” [18].

*Pocket money* when the individual said yes/no to question saying “did you have pocket money?” [19].

#### Data collection instrument and process

Data were collected using a semi-structured, pre-tested and self-administered questionnaire adapted from the literatures. The data collection tool was prepared in English and then translated into local language Amharic and finally returned to English by English language expertise. Four midwives were involved in the data collection process.

Appropriate information and instructions were given on the objective, the relevance of the study, confidentiality of information, respondent’s rights, informed consent, and technique of data collection and 1-day training was given to data collectors and the supervisor on data collection. Before the actual data collection, pre-test was done on 5% of students in Dejen secondary and preparatory school which was out of the study setting but nearby. The collected data checked for completeness and clarity by principal investigator and supervisor. Privacy and confidentiality of the respondents were maintained throughout the data collection period.

#### Data analysis

Data coded and entered into a computer using Epi info version 7.2.0.1 and checked for completeness and transferred to SPSS version 20 for analysis. Descriptive statistics like frequencies, percentage, proportion and mean computed. Bi-variate logistic regression used to identify variables that crudely associated and variables with p-values less than or equal to 0.05 fitted to multiple logistic regression. Then association between dependent and independent variables was assessed using adjusted odds ratio (AOR), 95% CI and *p* value of ≤ 0.05 considered statistically significant.

### Results

#### Socio-demographic characteristics

From the selected 624 school adolescents, a total of 600, adolescents aged between 10 and 19 completed the questionnaire while 24 refused to participate in the study, giving a response rate of 96.15%. Three hundred four (50.7%) of the respondents were females. The mean age was 17.31 years. The minimum and maximum ages were 15 and 18 years respectively. Five hundred eighty-three (97.2%) were Amhara in ethnicity (Table 1).

**Table 1:** Socio-demographic characteristics of adolescent students in Debre-Markos secondary and preparatory schools, from Nov. 23–27, 2017 (n = 600)

Variables	Frequency	Percent (%)
*Age*		
15–17	278	46.3
18	322	53.7
*Gender*		
Male	296	49.3
Female	304	50.7
*Current residence*		
Rural	297	49.5
Urban	303	50.5
*Grades*		
Grade 9	154	25.7
Grade 10	148	24.7
Grade 11	146	24.3
Grade 12	152	25.3
*Ethnicity*		
Amhara	583	97.2
Oromo	17	2.8
*Religion*		
Orthodox	565	94.1
Muslim	16	2.7
Protestant	19	3.2
*Attending church/mosque programs*		
Yes	581	96.8
No	19	3.2
*How often attend religious services*		
Every day	139	23.9
Every week	228	39.2
Every month	139	23.9
Every year	31	5.3
No response	44	7.6
*Pocket money*		
Yes	196	32.7
No	404	67.3

#### Parental characteristics

From the total participants, three-hundred thirty three (55.4%) responded that they were living with their mothers and fathers, 583 (97.2%) and 552 (92%) of respondents reported that their mothers and fathers were alive respectively. Two hundred sixty-four (44.3%) reported that they did not know their parents’ income and 263 (44.1%) participants responded this as greater than 53.6 USA dollars (Table 2).

**Table 2:** Parental characteristics of adolescent students in Debre-Markos secondary and preparatory school from Nov. 23–27, 2017 (n = 600)

Variables	Frequency	Percent (%)
*With whom usually live*		
Father and mother	333	55.4
Mother only	57	9.5
Father only	18	3.0
Relatives	43	7.2
Friends	22	3.7
Alone	127	21.2
*Parental residence*		
Rural	275	46.1
Urban	321	53.9
*Mother alive*		
Yes	583	97.2
No	17	2.8
*Maternal educational status*		
Unable to read and write	113	19.4
Read and write	168	28.8
Primary (1–8)	94	16.1
Secondary (9–10)	38	6.5
Above secondary	170	29.2
*Maternal occupational status*		
Housewife	216	37.0
Daily laborer	17	2.9
Farmer	174	29.8
Government employ	115	19.7
Merchant	25	4.3
Housemaid	36	6.2
*Father alive*		
Yes	552	92.0
No	48	8.0
*Paternal educational status*		
Unable to read and write	69	12.5
Read and write	168	30.4
Primary (1–8)	69	12.5
Secondary (9–10)	35	6.3
Above secondary	211	38.2
*Paternal occupational status*		
Daily laborer	22	4
Farmer	254	46
Merchant	99	17.9
Government employ	161	29.2
Other^a^	16	2.9
*Monthly income of parents*		
< 18.9 USA dollar	19	3.2
18.9–35.7 USA dollar	29	4.9
35.75–53.6 USA dollar	21	3.5
> 53.6 USA dollar	263	44.1
I don’t know	264	44.3
*Parents marital status*		
Currently married	495	82.5
Separated	30	5.0
Divorced	16	2.7
Widowed	42	7.0
Other^b^	17	2.8

^a^Priest

^b^Whose mother died

#### Habits of the respondents

Greater than two third, 413 (68.8%) of the participants responded that they were not alcohol users but 62 (10.3%) reported that they were khat users.

#### Sexual behavior of respondents

Two hundred forty seven (41.2%) of the participants responded that they usually watch films and magazines having sexual contents and nearly one third, 188 (31.3%) of the whole participants responded that they had girl/boyfriends and practiced sexual intercourse.

#### Reproductive health

From the total respondents, only 95 (15.8%) participants responded that they had sexual issue discussion with their parents. Three hundred seventeen (52.8) responded that they had sexual issue discussion with their close friends and 72 (12%) were pressured to have sexual intercourse.

#### Associated factors of premarital sexual a intercourse

Crudely associated variables were: sex, current residence, pocket money, with whom usually live, mother alive, father alive, place of parent’s live, discussion the sexual issue with close friends and peer pressure.

Independently and positively associated variables in adjusted analysis were: being male, having pocket money, adolescents who did not discuss the sexual issue with close friends and Peer pressure (Table 3).

**Table 3:** Bivariable and multivariable analysis of factors associated with premarital d sexual intercourse among adolescent students in Debre-Markos secondary and preparatory school from Nov. 23–27, 2017 (n = 600)

Variables	Premarital sex	COR (95% CI)	AOR (95% CI)
	*Yes (%)*		
*Sex*			
Male	123 (20.5)	2.61 (1.83, 3.74)**	1.88 (1.21, 2.93)**
Female	65 (10.8)	1	1
*Current residence*			
Rural	105 (17.5)	1.45 (1.03, 2.05)*	
Urban	83 (13.8)	1	
*Pocket money*			
Yes	100 (16.7)	3.74 (2.59, 5.39)**	3.07 (1.96, 4.81)***
No	88 (14.7)	1	1
*With whom usually live*			
Father and mother	79 (13.2)	1	
With my mother only	21 (3.5)	1.88 (1.03, 3.39)*	
With my father only	10 (1.7)	4.02 (1.53, 10.53)**	
With my relatives	8 (1.3)	0.73 (0.327, 1.65)	
With my friends	10 (1.7)	2.68 (1.11, 6.44)*	
Alone	60 (10)	2.88 (1.87, 4.43)**	
*Mother alive*			
Yes	177 (29.5)1	1	
No	1 (1.8)	4.20 (1.53, 11.55)**	
*Father alive*			
Yes	157 (26.2)	1	
No	31 (5.2)	4.59 (2.47, 8.53)**	
*Place of parents live*			
Rural	99 (16.6)	1.49 (1.05, 2.11)*	
Urban	88 (14.8)	1	
*Discuss sexual issue with close friends*			
Yes	42 (7)	1	1
No	146 (24.3)	6.98 (4.68, 10.41)**	8.56 (5.27, 13.91)***
*Peer pressure*			
Yes	56 (9.3)	10.50 (5.82, 18.93)**	7.65 (3.73, 15.69)***
No	132 (22)	1	1

* p-value < 0.05, ** p-value < 0.01 and *** p-value < 0.001

### Discussion

Proportion of adolescents who had premarital sex was 31.3%, 95% CI (27.3, 34.8). It was in line with the study from Bahir-Dar, 30.8% [10], Maichew, 29.3% [5], Gondar and Meteme, 31.9% [13].

But this study’s finding was lower than the study from Nepal, 36.5% [20]. This disparity could be justified by the difference in a background of the study participants and variation in the study areas.

Also, this study’s finding was higher than a study from Shendi, 19% [14], Shire-Endasellassie, 19% [9], Alamata, 21.1% [21] and Jimma, 25.3% [17]. This could be explained by decrement of discussion about reproductive health risks and rise of peer pressure.

One of the predictor variables in this study was sex. It shows that male students were more engaged for premarital sexual intercourse (AOR = 1.9, 95% CI 1.21, 2.9). It was consistent with studies from Yabello [15], Bahir Dar [22] and Malaysia [23]. It might be due to males have more freedom in sexual engagement than females.

The other variable that was positively associated with premarital sexual intercourse was having pocket money. It triggers adolescent student to have sexual intercourse (AOR = 3, 95% CI 1.9, 4.8). It was similar with the studies from Arba Minch [24] and Jimma [19]. It could be due to financially equipped students are likely to drink alcohol, to date their opposite friends and to buy the porn films that all can trigger for sexual intercourse.

And also, the other positive predicator variable for premarital sexual intercourse was having discussions with close friends. Those who did not have discussions with their close friends were more engaged with premarital sexual intercourse (AOR = 8.6, 95% CI 5.28, 13.9). It could be explained as those who have discussions are likely to be knowledgeable about premarital sexual intercourse risks including HIV transmission which can make adolescent students to abstain from sexual intercourse.

The additional positive predictor variable for premarital sexual intercourse was peer pressure. Those who were pressurized by their friends were more engaged with premarital sexual intercourse (AOR = 7.7, 95% CI 3.7, 15.7). It was consistent with studies from Bahir-Dar [10], Maichew [5], Alamata [21], Gondar and Metema [13]. It could be due to the fact that peers in adolescents is an important factor to influence personality and behavior changes.

## Limitations

It was good if the data collection tools were triangulated with the qualitative data collection techniques like in-depth interview.

## Abbreviations

AOR: adjusted odds ratio
CI: confidence interval
COR: crude odds ratio
STI: sexually transmitted infection
WHO: World Health Organization

## References

[1] Berihun H (2014). Assessment of the prevalence of premarital sex and unprotected sexual practices among secondary school adolescent students in Sebeta town, Oromia regional state, Ethiopia.

[2] Mengistie Z, Wolie E, Abawa E, Ebre E, Adera A (2015). Knowledge attitude and practice towards premarital sex and HIV/AIDS among Mizan-Tepi University students, south west Ethiopia. Sci J public Health.

[3] World Health Organisation (1999). Programming for adolescent health and development. Report of a WHO/UNFPA/UNICEF Study Group on Programming for Adolescent Health.

[4] UNICEF. Adolescence and youth. 4th ed. Colorado: Harper Collins Publisher; 2016. http://www.unicef.org/adolescence/.

[5] Salih NA, Metaferia H, Reda AA, Biadgilign S (2015). Premarital sexual activity among unmarried adolescents in northern Ethiopia: a cross-sectional study. Sex Reprod Healthcare..

[6] Chandra-Mouli V, Camacho AV, Michaud PA. WHO guidelines on preventing early pregnancy and poor reproductive outcomes among adolescents in developing countries. J Adolesc Health. 2013;52(5):517–22 http://ovidsp.ovid.com/ovidweb.cgi?T=JS&PAGE=reference&D=medl&NEWS=N&AN=23608717%5Cn. 10.1016/j.jadohealth.2013.03.002 23608717

[7] Regassa T, Chala D, Adeba E (2016). Premarital sex in the last twelve months and its predictors among students of Wollega University, Ethiopia. Ethiop J Health Sci..

[8] Teferra TB, Erena AN, Kebede A (2015). Prevalence of premarital sexual practice and associated factors among undergraduate health science students of Madawalabu university, Bale Goba, south east Ethiopia: institution based cross sectional study. Pan Afr Med J..

[9] Ayalew A, Abreha K, Shumey A, Berhane K (2015). Development magnitude and predictors of early sexual debut among high and preparatory school students in northern Ethiopia : a school-based cross-sectional study. J Health Educ Res.

[10] Mulugeta Y, Berhane Y (2014). Factors associated with pre-marital sexual debut among unmarried high school female students in Bahir Dar town, Ethiopia: cross-sectional study. Reprod Health..

[11] Central Statistical Agency Addis Ababa E, ICF TDP, Rockville, Maryland U. Ethiopia Demographic and Health Survey key indicators. 2016.

[12] Daba B. Assessment of premarital sexual practices and factors related to it among Ambo high school students. 2016. http://etd.aau.edu.et/handle/123456789/8550.

[13] Terfe A, Jibril S, Laddunuri MM (2016). Magnitude of sexual debut and associated factors among high school girl students in Amhara region, north western Ethiopia. Econ Affairs.

[14] Bogale A, Seme A. Premarital sexual practices and its predictors among in-school youths of shendi town, west Gojjam zone, North Western Ethiopia. Reprod Health [Internet]. 2014;11(1):49. Available from: http://www.scopus.com/inward/record.url?eid=2-s2.0-84902909679&partnerID=tZOtx3y1. 10.1186/1742-4755-11-49 PMC4081458 24961239

[15] Beyene AS, Seid AM (2014). Prevalence of premarital sex and associated factors among out-of-school youths (aged 15–24) in Yabello town, Southern Ethiopia: a community based cross-sectional study. Adisu Shunu Beyene and Abdulbasit Musa Seid..

[16] Wikipidia. Debre-Marqos. In: free encyclopedia. 2017.

[17] Taye A, Asmare I. Prevalence of premarital sexual practice and associated factors among adolescents of Jimma Preparatory School Oromia Region, south west Ethiopia. J Nurs Care. 2016;5(2). http://www.omicsgroup.org/journals/prevalence-of-premarital-sexual-practice-and-associated-factors-amongadolescents-of-jimma-preparatory-school-oromia-region-south-w-2167-1168-1000332.pdf.

[18] Meleko A, Mitiku K, Kebede G, Muse M, Moloro N. Magnitude of pre-marital sexual practice and its associated factors among Mizan Preparatory School Students in Mizan Aman Town, south west Ethiopia. J Community Med Health Educ. 2017;7(4). https://www.omicsonline.org/open-access/magnitude-of-premarital-sexual-practice-and-its-associated-factors-amongmizan-preparatory-school-students-in-mizan-aman-town-south-2161-0711-1000539.php?aid=91601.

[19] Hurissa BF, Tebeje B, Megersa H. Prevalence of Pre-marital Sexual Practices and Associated Factors among Jimma Teacher Training College Students in Jimma Town, south west Shoa Zone, Oromiya Region, Ethiopia-2013. J Womens Heal Care. 2015;4(1):1–10. http://www.omicsgroup.org/journals/prevalence-of-premarital-sexual-practices-and-associated-factors-among-jimma-teacher-training-college-2167-0420.1000221.php?aid=36489.

[20] Adhikari R, Adhikari RP, Maharjan RK. Masturbation practice among school adolescents in Makawanpur District, Nepal. Int J Res. 2015;2(6):323–30. http://edupediapublications.org/journals/index.php/ijr/article/view/2207.

[21] Kassa GM, Woldemariam EB (2014). Prevalence of premarital sexual practice and associated. Global J Med Res..

[22] Taye B, Nurie T (2017). Assessment of premarital sexual practices and associated factors among private college regular students in Bahir dar city, northwest Ethiopia : a cross-sectional study. Int J Hort Agric Food Sci.

[23] Nawi AM, Roslan D, Idris IB, Hod R (2017). Bullying and truancy: predictors to sexual practices among school-going adolescents in Malaysia—a cross-sectional study. Med J Malaysia.

[24] Sorato MM BZ. Magnitude and predictors of premarital sexual practice among unmarried undergraduate students, at Arba Minch University, Ethiopia, 2015. Int J Reprod Fertil Sex Health. 2017;4(2):95–104. http://scidoc.org/articlepdfs/IJRFSH/IJRFSH-2377-1887-04-201.pdf.

